# Supplementary material for: Levels of Physical Activity in Children with Extremity Fractures a Dutch Observational Cross-Sectional Study
Source: Children (Basel). 2022 Mar 1;9(3):325. doi: 10.3390/children9030325 (PMC8947536; doi:10.3390/children9030325)
Supplement: Supplementary file 1 [file children-09-00325-s001.zip › children-1608180-supplementary.pdf]

Supplementary File S1

1) General and medical questions

1. What is your child's gender?
    - Boy
    - Girl

2. What age is your child? …

3. Which emergency department did your child visit?
    - CWZ
    - Radboudumc

4. Was your child at the Emergency Department of Radboudumc/CWZ without a parent/guardian?
    - Yes
    - No

5. What is your child's weight (in kilograms) ? …

6. What is your child's length (in centimeters)? …

7. Does your child have any medical condition for which check-ups with a specialist are required?
    - Yes, proceed to question 8
    - No, proceed to question 9

8. What medical condition does your child have?
If the option is not listed answer "Other condition, namely…" and fill in what condition it concerns.
    - Asthma
    - Diabetes Mellitus
    - Botdysplasia
    - Other condition, namely …

9. Does your child use medication(s)?
    - No
    - Yes, namely…

2) Questions on the type of fracture, cause and conditions

1. What caused your child's fracture?
   - o A fall of more than 5 meters
   - o A car crashes with more than 45 km per hour against a stationary object (for instance: a tree, pedestrian, or car)
   - o A collision with a pedestrian with a velocity of >10km/h
   - o Was thrown out of a vehicle or sitting in a vehicle that overturned
   - o Fell of a horse
   - o None of these events

2. How many fractures did you child have during the visit to the Emergency Department?
   - o One
   - o Two
   - o Three
   - o Four or more

3. What part of the body was the fracture located?
   Cross the body parts that are applicable. Multiple options are possible.
   - o Thorax
   - o Face or skull
   - o Pelvis
   - o Upper extremities: hand, arm, elbow, shoulder, collarbone
   - o Lower extremities: foot, knee or leg

4. What caused the fracture?
   Choose the option most applicable for the situation.
   - o A fall of less than 0.5 meter
   - o While practicing a sport
   - o A fall of 0.5 to 3 meters
   - o A fall of more than 3 meters

5. Can you describe what happened when the fracture was originated?

____

____

____

____

____

From now on, the questions will concern the period ***before*** the incident that caused the fracture.

3) Physical activity

Take a normal week of the past few months prior to the event that caused the fracture into account. Could you indicate the days per week your child participated in the below mentioned activities. Also, how much time did he or she spend on the mentioned activity. Lastly, indicate what the effort the activity required.

Residential/school activities

	amount of days per week	average time per day	effort
a. walking from/to school	☐ days	☐☐ hour ☐☐ min.	☐ slow<br>☐ average<br>☐ fast
b. biking from/to school	☐ days	☐☐ hou r ☐☐ min.	☐ slow<br>☐ average<br>☐ fast
c. not applicable	☐		

Physical activity at school

	amount of hours per week
a. Light to moderate intensity physical activity at school<br>(for example, playing outside during lunch break)	☐☐
b. Moderate to intense physical activity at school<br>(for example, physical education)	☐☐ hour
c. not applicable	☐

Leisure time

	amount of days per week		average time per day				effort
a. walking and biking (apart from school time)	☐	days	☐☐	hour	☐☐	min.	☐ slow<br>☐ average<br>☐ fast
b. playing outside (apart from school time)	☐	days	☐☐	hou r	☐☐	min.	☐ slow<br>☐ average<br>☐ fast

c. not applicable ☐

Sports

For example, tennis, handball, gymnastics, fitness, ice-skating, swimming, judo. You can fill out 4 sports maximum. Choose in the case of more than 4 sports the most intensive sports.

		amount of days per week	average time per day		effort
a.	☐	☐ days	☐☐ hour	☐☐ min.	☐ slow<br>☐ average<br>☐ fast
b.	☐	☐ days	☐☐ hour	☐☐ min.	☐ slow<br>☐ average<br>☐ fast
c.	☐	☐ days	☐☐ hour	☐☐ min.	☐ slow<br>☐ average<br>☐ fast
d.	☐	☐ days	☐☐ hour	☐☐ min.	☐ slow<br>☐ average<br>☐ fast

Total

On average how many days per week was your child performing at least thirty minutes of biking, walking, gymnastics, sports, or any other mentioned activity?

Add all the activities to calculate the amount of days.

On average ________ days.

4) Screen time

Take a normal week of the months *prior to the fracture* into account.

Media devices & television

1. Which of the follow media devices did your child use? Fill “none of the options” in case none of the options are applicable.
   - o Television without direct internet access
   - o Television with direct internet access (Smart TV or Connected TV)
   - o Smartphone
   - o Mobile Phone without internet access
   - o Desktop computer (Personal Computer, also iMac)
   - o Laptop or netbook
   - o Tablet (e.g. iPad or Android PC tablet)
   - o E-reader
   - o Game console (e.g. Xbox, Playstation, Wii, PSP)
   - o Mediaplayer (e.g. iPod, mp3-speler)
   - o Mediacenter or dongle on the TV (e.g. Apple TV, Google TV)
   - o Video-, DVD, Blu Ray- or Harddiskrecorder
   - o Wearables (e.g. SmartWatch and Google Glass)
   - o None of the options

2. Does your child watch television?
   - o Yes
   - o No

3. How many days a week does your child watch television?
   Fill out the average days a week.
   - o One
   - o Two
   - o Three
   - o Four
   - o Five
   - o Six
   - o Every day of the week

4. On the days that your child does watch television, how much time was spent per day?

		hour			min.

Internet

The next questions are about the use of internet. With internet use the following is meant: the visitation of websites and use of apps. Internet can be used at the multiple devices such as a computer, laptop, tablet or Smartphone.

1. Does your child use the internet?
    - o Yes
    - o No

2. How many days a week did your child use the internet? ☐

3. On the days your child used the internet, how much time was spent per day?

☐☐ hour ☐☐ min.

Games and online games

The next questions are about gaming and online gaming. The media devices on which games can be played are for example X-Box, Gameboy or Wii. Examples of online games are World of Warcraft or League of Legends.

1. Does your child play (online) games?
    - o Yes
    - o No

2. How many days per week did your child play (online) games? ☐

3. On the days that your child played (online) games, how much time was spent per day?

☐☐ hour ☐☐ min.

This is the end of the questionnaire. Thank you very much for your participation.
